# Assessment of Electrolyte Imbalances as an Early Manifestation of Refeeding Syndrome in Critically Ill Patients Receiving Total Parenteral Nutrition at a Tertiary Hospital in Mexico City

**DOI:** 10.7759/cureus.101608

**Published:** 2026-01-15

**Authors:** Alejandro Martinez-Esteban, Sofía Peña-Solórzano, Erika Paola Sanchez-Saavedra, Miguel M Rojas-Evaristo, Victor M de la Puente-Diaz de Leon

**Affiliations:** 1 General and Gastrointestinal Surgery, Hospital Medica Sur, Mexico City, MEX; 2 General and Gastrointestinal Surgery, Facultad Mexicana de Medicina, Universidad La Salle, Mexico City, MEX; 3 Internal Medicine, Hospital Medica Sur, Mexico City, MEX; 4 Critical Care Medicine, Hospital Medica Sur, Mexico City, MEX

**Keywords:** critical care, critically ill patients, electrolyte imbalances, parenteral nutrition, refeeding syndrome

## Abstract

Introduction

The nutritional status of critically ill patients is a key determinant of their clinical outcomes. Total parenteral nutrition (TPN) is an essential intervention when oral or enteral routes are not feasible, but it carries important risks such as hepatic and infectious complications, and particularly the development of refeeding syndrome (RFS), a potentially life-threatening condition associated with severe metabolic disturbances.

Objectives

The primary objective of the study was to determine the primary electrolyte imbalances associated with the development of RFS in critically ill patients receiving TPN. Secondary objectives were to determine the prevalence of RFS and evaluate the most effective prevention strategies in this population.

Methods

We conducted a retrospective descriptive study including critically ill hospitalized adults receiving TPN at Hospital Medica Sur, Mexico City, Mexico, between December 2024 and July 2025. Of 1,433 screened patients, 195 met the inclusion criteria. Clinical and laboratory data were collected from medical records with a seven-day follow-up after initiation of TPN. Outcomes studied were electrolyte disturbances (hypophosphatemia, hypokalemia, and hypomagnesemia) and prevalence of diagnosis of RFS using National Institute for Health and Care Excellence (NICE) diagnostic criteria. Logistic regression analyses adjusted for confounders were performed to identify associated factors (age, sex, BMI, significant weight loss, TPN duration, and nutritional risk with NRS-2002). R software (R Foundation for Statistical Computing, Vienna, Austria) was used for statistical analyses, with p < 0.05.

Results

A high prevalence of electrolyte imbalances was observed. Hypokalemia was documented in 37% of the study population (n = 73), hypophosphatemia in 36% (n = 71), and hypomagnesemia in 24% (n = 47). A dose-response relationship was identified: compared with patients without electrolyte alterations, the risk of RFS progressively increased with the presence of one (OR = 1.39, 95% CI: 1.27-1.52), two (OR = 2.54, 95% CI: 2.31-2.79), and three imbalances (OR = 2.55, 95% CI: 2.32-2.81).

Conclusions

RFS is a common complication in critically ill patients receiving TPN. The presence of electrolyte imbalances is directly and significantly associated with the development of this syndrome; Therefore, early identification allows the implementation of preventive interventions aiming at reducing the morbidity and mortality associated with nutritional support.

## Introduction

The nutritional status of an individual is a multifactorial concept encompassing factors such as caloric intake, absorption, nutrient utilization, body composition, and overall health. Thus, there is a need to determine inadequate or excessive intakes to design interventions that improve health and prevent diet-related diseases [1].

The Nutritional Risk Screening-2002 (NRS-2002) is a nutritional assessment system used to identify patients at risk of malnutrition who may require dietary or nutritional interventions. [1,2]. It evaluates three main components: weight loss, body mass index (BMI), and food intake. A higher score indicates greater nutritional risk and the need for intervention [2].

Total parenteral nutrition (TPN) refers to the administration of dextrose, lipids, amino acids, electrolytes, vitamins, minerals, and trace elements via intravenous parenteral route [3-5]. It is a lifesaving intervention for patients who are unable to receive oral or enteral nutrition. Despite its benefits, this treatment is costly and carries a risk of hepatobiliary, infectious, and electrolyte complications [6-8].

In general, half of the daily requirements are administered on the first day and, if tolerated without uncontrolled hyperglycemia, sudden electrolyte changes, or lipid abnormalities, the full target is reached on the second day [9-11]. TPN must be prescribed daily due to the potential for shifting electrolyte imbalances. In general, oral feeding is preferred over enteral or parenteral support; however, patient comorbidities may affect this decision. In the intensive care unit (ICU), most patients are unable to receive enteral feeding due to comorbidities, clinical condition, and other causes, primarily related to alterations in gastrointestinal function [12-14].

Refeeding syndrome (RFS) is a potentially life-threatening condition resulting from rapid shifts in fluids and electrolytes when malnourished patients receive oral, enteral nutrition, or TPN [15]. The first cases were reported in the 1940s, when a series of World War II prisoners suffering from severe malnutrition were found to develop cardiovascular and neurological manifestations after they had been repleted with nutrients following prolonged starvation [16]. The first systematic review addressing refeeding syndrome was published by Friedli et al. in March 2017, focusing on its definition and the development of preventive and treatment strategies. It is considered a life-threatening condition that involves severe metabolic abnormalities, such as impaired glucose metabolism, electrolyte imbalances (hypophosphatemia, hypokalemia, hypomagnesemia), and thiamine deficiency [17]. The primary aim of this study was to determine the primary electrolyte imbalances associated with the development of RFS in critically ill patients receiving TPN.

## Materials and methods

This was a retrospective descriptive study conducted at Hospital Medica Sur, Mexico City, Mexico, from December 2024 to July 2025. Every individual admitted as a patient to the hospital signed a privacy notice authorizing the use of their data for research purposes. The study was approved by the Research Ethics Committee of Médica Sur (approval number: 2024-EXT-922).

Objectives

The primary objective of the study was to determine the primary electrolyte imbalances associated with the development of RFS in critically ill patients receiving TPN. Secondary objectives were to determine the prevalence of RFS and evaluate the most effective prevention strategies in this population

Study population

The study inclusion criteria were adult patients over 18 years of age hospitalized in critical condition and at risk of malnutrition. The exclusion criteria were patients hospitalized in the Gynecology and Obstetrics area, patients in palliative care, and any other patient who did not require TPN.

**Figure 1:**
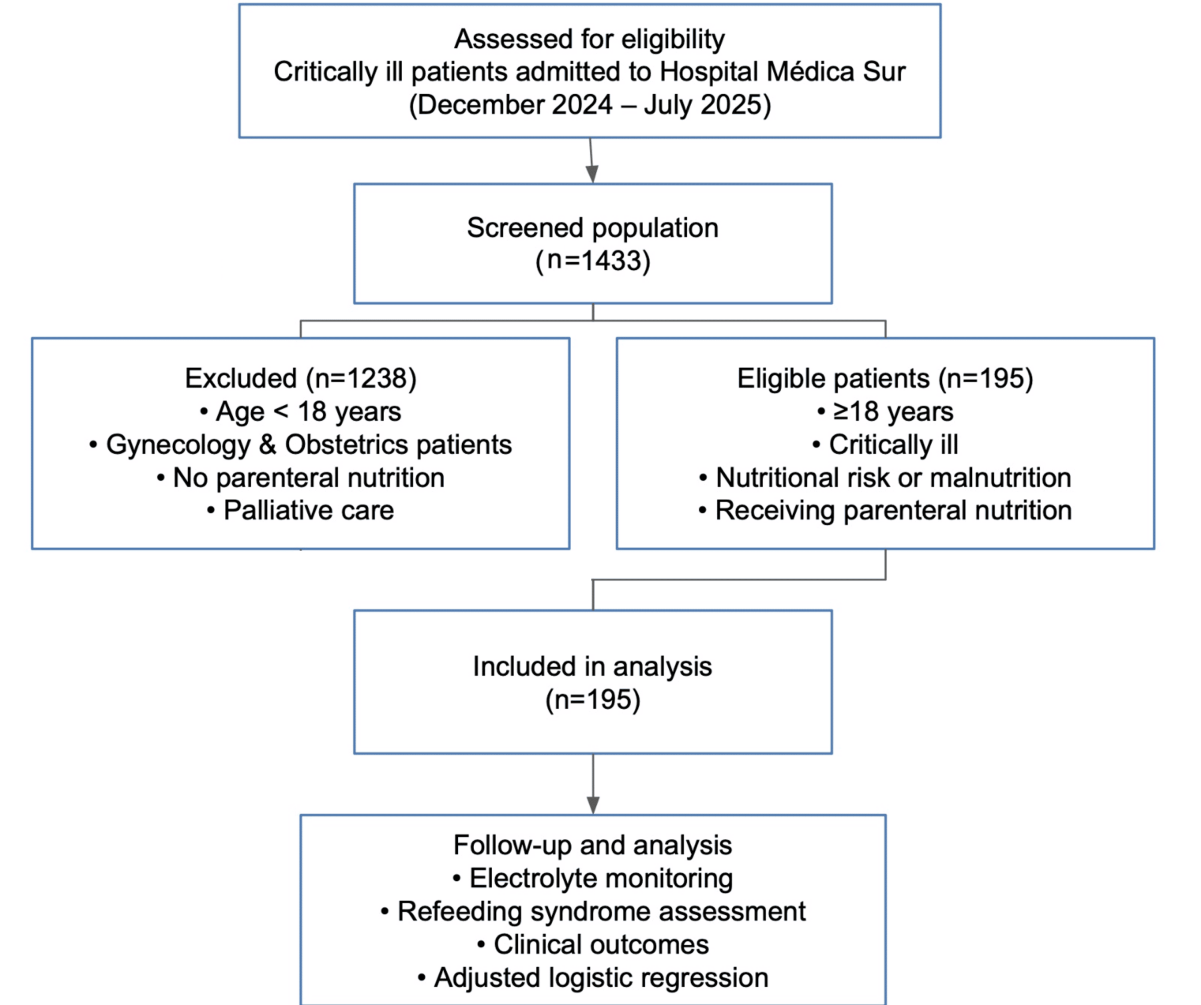
Cohort flow diagram of patient selection and inclusion in the study Patient eligibility was assessed, and data were acquired from 1433 critically ill adult patients admitted to the hospital between December 2024 and July 2025. Of these, 1238 patients were excluded, and 195 patients who met all criteria were included in the final cohort. The presence of electrolyte imbalances and the occurrence of refeeding syndrome were assessed, and the results were analyzed using adjusted logistic regression models.

The study population consisted of 1433 critically ill adult patients hospitalized at the hospital. Of these, 1238 patients were excluded for not meeting the inclusion criteria, and 195 patients who met all the criteria were included in the final cohort (n = 195). Data were obtained from medical records and electronic health records with clinical and laboratory follow-up within seven days after the start of TPN. The reference range for laboratory values ​​for electrolyte imbalances comes from the ranges established by the laboratory and Clinical Pathology service of the hospital. Figure 1 shows the flow chart for the selection of participants.

Data collection

The independent variables considered in the study include: sex (female and male), age (years), reason for admission to ICU (shock, desaturation, bleeding, etc.), comorbidities (pre-existing conditions), and severity criteria (low, high, very high risk). The dependent variables are: electrolyte imbalance (hypophosphatemia, hypokalemia, and hypomagnesemia), diagnosis of RFS according to the NICE Criteria Guidelines [18] (yes or no), and clinical outcomes. The information was collected directly from clinical records and organized in a database designed specifically for this study.

Data analysis

The clinical and laboratory evolution of the patients was evaluated within the first seven days, documenting the development of RFS, the interventions performed to prevent its progression, and each patient's outcome. This information allowed the identification of modifiable risk factors and the strengthening of strategies for the prevention and timely management of RFS in critically ill patients.

Quantitative variables were summarized by the median (interquartile range (IQR)), and qualitative variables by frequencies and percentages. All analyses were conducted using R statistical software version 4.3.2 (R Foundation for Statistical Computing, Vienna, Austria, https://www.R-project.org/). A value of P < 0.05 was considered to be statistically significant. To evaluate the association between electrolyte imbalances (hypophosphatemia, < 3.2 mg/dL, reference interval: 3.2-4.6 mg/dL; hypokalemia, < 3.9 mg/dL, reference interval: 3.9-5.3 mg/dL; hypomagnesemia, < 1.8 mg/dL, reference interval: 1.8-2.5 mg/dL) and the development of RFS according to the NICE Criteria Guidelines [18].

Given that one of the primary dependent variables was binary (presence or absence of electrolyte imbalance), a logistic regression analysis was performed to identify factors that increase or decrease the probability that a patient at risk will develop an electrolyte imbalance during TPN administration. Binary logistic regressions were performed, adjusted for the following confounding variables: age, sex, BMI, significant weight loss, NRS-2002 score, duration of TPN, and nutritional risk.

## Results

A total of 195 patients with severe illness were recruited from December 2024 to July 2025; 50% of the patients were men (n = 98) and 50% women (n = 97), with a median age of 67 years (IQR: 52-76) (Table 1); thus representing mainly older adults, a patient category prone to comorbidities, and in-hospital nutritional decline. Regarding anthropometric characteristics, the median weight was 65 kg (IQR: 57-74), and the BMI was 23.3 kg/m² (IQR: 21.0-26.7). When classifying patients according to BMI, most were within the normal weight range at 55% (n = 108), followed by overweight at 28% (n = 55), obesity at 9% (n = 18), and malnutrition at 7% (n = 14). This result is interesting, as there may be nutritional deficiencies in the obese patient.

**Table 1:** Clinical and demographic characteristics values Continuous values are expressed as median (interquartile range (IQR)); categorical variables are expressed as absolute number and percentage.

Parameter	Values
Age (years), median (IQR)	67 (IQR: 52–76)
Sex, n (%)	
Male	98 (50%)
Female	97 (50%)
Weight (kg), median (IQR)	65 (IQR: 57–74)
Height (meters), median (IQR)	1.65 (IQR: 1.60, 1.73)
BMI (kg/m²), median (IQR)	23.3 (IQR: 21.0–26.7)
Nutritional Classification, n (%)	
		Malnutrition	14 (7.2%)
				Normal weight	108 (55%)
						Overweight	55 (28%)
								Obesity	18 (9.2%)

Table 2 shows the associated comorbidities. Hypertension was the most prevalent at 30% (n = 59), followed by type 2 diabetes at 18% (n = 35), thyroid disorders at 13% (n = 25), and dyslipidemia at 11% (n = 22). CKD was identified in 4.6% of patients (n = 9), most frequently in the. advanced stages of CKD (Grade 3-5). Various cardiovascular histories were noted, including atrial fibrillation (4.6%, n = 9) and ischemic heart disease (5.1%, n = 10), as well as several pulmonary conditions, mainly chronic obstructive pulmonary disease (COPD) (3.1%, n = 6). Liver cirrhosis was documented in 2.6% of patients (n = 5). Any oncologic disease was reported in 39% of patients (n = 76), predominantly colon cancer (8.2%, n = 16), lung cancer (4.1%, n = 8), pancreatic cancer (6.2%, n = 12), and hematologic malignancies (4.6%, n = 9). Other comorbidities included deep vein thrombosis (DVT) and pulmonary embolism (PE) in 9.2% of patients (n = 18) and autoimmune diseases, the most frequent being rheumatoid arthritis at 4% (n = 8). Up to 41% of the sample (n = 80) reported other comorbidities, including osteoporosis, benign prostatic hyperplasia, gout, gastroesophageal reflux disease, peptic ulcer disease, and irritable bowel syndrome.

**Table 2:** Associated comorbidities (N = 195) CKD: chronic kidney disease; COPD: chronic obstructive pulmonary disease; DVT: deep vein thrombosis; PE: pulmonary embolism

Comorbidities	Frequency (Percentage)
Cardiopathies	34 (17.4%)
Atrial fibrillation	9 (4.6%)
Ischemic heart disease	10 (5.1%)
Valvular heart disease	1 (0.5%)
Heart failure	6 (3.1%)
Others	8 (4.1%)
Hepatic cirrhosis	5 (2.6%)
Type 2 diabetes	35 (18%)
Dyslipidemia	22 (11%)
Thyroid disorders	25 (13%)
Autoimmune diseases	14 (7.2%)
Systemic lupus erythematosus	1 (0.5%)
Rheumatoid arthritis	6 (3.1%)
Coagulation disorders	2 (1.0%)
Others	5 (2.6%)
CKD	9 (4.6%)
No CKD	186 (95%)
Stage 3	5 (2.6%)
Stage 4	1 (0.5%)
Stage 5	3 (1.5%)
Neurological diseases	17 (8.7%)
Ischemic stroke	7 (3.6%)
Hemorrhagic stroke	2 (1.0%)
Others	8 (4.1%)
Oncologic disease	76 (39%)
Pulmonary	8 (4.1%)
Breast	6 (3.1%)
Uterus and adnexa	1 (0.5%)
Liver	2 (1.0%)
Colon	16 (8.2%)
Kidney	1 (0.5%)
Pancreas	12 (6.2%)
Hematologic	9 (4.6%)
Prostate	7 (3.6%)
Others	36 (18%)
Hypertension	59 (30%)
Pulmonary diseases	13 (6.7%)
COPD	6 (3.1%)
Asthma	3 (1.5%)
Others	4 (2.1%)
DVT/PE	18 (9.2%)
Other comorbidities	80 (41%)

Upon admission to the hospital, more than half of the patients (52%, n = 102) reported significant weight loss, while 68% (n = 133) presented with loss of appetite. The median score on the NRS 2002 scale was 5.0 (IQR: 3.0-5.0), classifying 72% (n = 140) as being at high nutritional risk. The high prevalence of malnutrition in patients with obesity highlights the importance of systematic nutritional assessment upon hospital admission for the assessment of nutritional risk using the NRS 2002 score (Table 3). The median duration of TPN (personalized or pre-formed) was eight days (IQR: 6-14). The main indications for initiating TPN were prolonged fasting (35%, n = 69), low enteral intake (34%, n = 66), and during the postoperative period (26%, n = 50). Almost half of the patients (49%, n = 96) received commercial pre-formed TPN with a median caloric intake of 1135 kcal (IQR: 1035-1509).

**Table 3:** Nutritional characteristics, electrolyte imbalances and clinical outcomes of critically ill patients who received TPN (N=195) Continuous values are expressed as median (interquartile range); categorical variables are expressed as absolute number and percentage TPN: total parenteral nutrition; NRS-2002: Nutrition Risk Screening 2002; RFS: refeeding syndrome

Parameter	Value
Weight loss	102 (52%)
Loss of Appetite	133 (68%)
NRS-2002 (Points), median (IQR)	5 (3, 5)
Nutritional risk, n (%)	
High	140 (72%)
Moderate	53 (27%)
Low	2 (1.0%)
Days with TPN, median (IQR)	8 (6, 14)
Indication for TPN, n (%)	
		Fasting	69 (35%)
		Low enteral intake	66 (34%)
		Surgery	50 (26%)
		Malnutrition	10 (5.1%)
Pre-formed TPN, n (%)	96 (49%)
Caloric intake (kcal), median (IQR)	1,135 (1,035–1,509)
Electrolyte imbalance, n (%)	
Hypophosphatemia	71 (36%)
Hypokalemia	73 (37%)
Hypomagnesemia	47 (24%)
Any electrolyte imbalance	95 (49%)
Cumulative number of electrolyte imbalance , n (%)	
0	100 (51%)
1	33 (17%)
2	28 (14%)
3	34 (17%)
Arrhythmias, n (%)	
Supraventricular tachycardia	5 (2.6%)
Ventricular tachycardia	1 (0.5%)
Atrioventricular block	1 (0.5%)
Atrial fibrillation	1 (0.5%)
Use of Multivitamin Solution	96 (49.1%)
Clinical outcomes, n (%)	
Development of RFS	73 (37%)
Clinical outcome, n (%)	
Death	35 (18%)
Discharge	160 (82%)
Hospital readmission	43 (22%)

**Figure 2:**
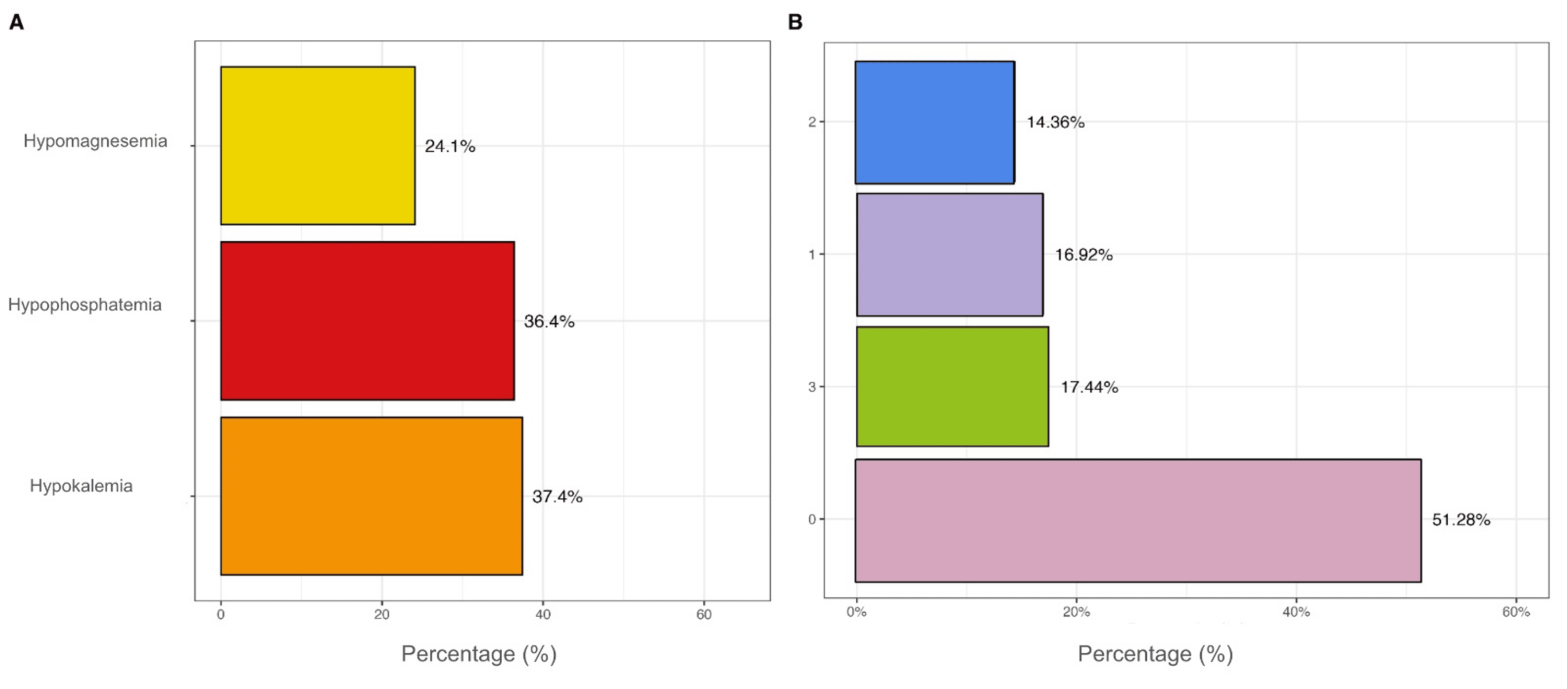
Presentation of electrolyte imbalances This figure describes the most common electrolyte imbalances (yellow: hypomagnesemia, red: hypophosphatemia and orange: hypokalemia) (2A), as well as the total electrolyte imbalances presented in the study (purple: 1 imbalance, blue: 2 imbalances, green: three imbalances, pink: no imbalance) (2B).

The reported prevalence of electrolyte imbalances was high. Hypokalemia was documented in 37% of the study population (n = 73), hypophosphatemia in 36% (n = 71), and hypomagnesemia in 24% (n = 47) (Figure 2A). Regarding the cumulative number of disorders, 1/3 of patients had more than one imbalance, while 51.3% (n = 100) showed no manifestations. The concurrence of all three disorders affected 17.4% (n = 34) (Figure 2B).

Arrhythmias were rare, occurring in only 4.1% (n = 8) of patients. The study identified the use of commercial multivitamin solution in 49.1% (n=96). Finally, regarding clinical outcomes, 37% (n = 73) developed RFS, 18% (n = 35) died during hospitalization, and 22% (n = 43) were readmitted after discharge.

**Figure 3:**
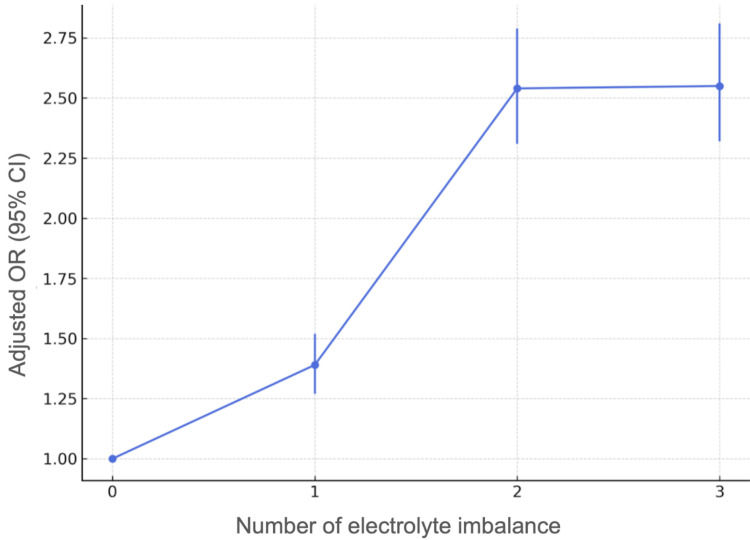
Increased risk of RFS with the number of electrolyte imbalances developed RFS: refeeding syndrome

The development of the three electrolyte imbalances was statistically significantly associated with a higher probability of presenting RFS. In the adjusted models, hypophosphatemia increased this probability by 2.26 times (95% CI: 2.09-2.45), hypokalemia by 1.93 times (95% CI: 1.75-2.14), and hypomagnesemia by 1.83 times (95% CI: 1.61-2.10). The presence of any imbalance doubled the risk of developing this syndrome (OR = 2.02; 95% CI: 1.85-2.20) (Table 4). In addition, a dose-response relationship was evident, as compared to patients without alterations, the risk of RFS increased progressively with the presence of one (OR = 1.39, 95% CI: 1.27-1.52), two (OR = 2.54, 95% CI: 2.31-2.79), and three imbalances (OR = 2.55, 95% CI: 2.32-2.81) (Figure 3).

**Table 4:** Association between electrolyte imbalances and the development of RFS in critically ill patients receiving TPN Adjusted models include age, sex, BMI, Significant weight loss, NRS 2002 score, days of TPN use, and risk of malnutrition. OR: odds ratio; 95% CI: 95% confidence interval; Ref: reference category; RFS: refeeding syndrome; TPN: total parenteral nutrition

Parameter	Unadjusted Models			Adjusted Models		
	OR	95% CI	p value	OR	95% CI	p value
Hypophosphatemia	2.34	2.17, 2.53	<0.001	2.26	2.09, 2.45	<0.001
Hypokalemia	2.04	1.85, 2.26	<0.001	1.93	1.75, 2.14	<0.001
Hypomagnesemia	1.98	1.75, 2.25	<0.001	1.83	1.61, 2.10	<0.001
Any electrolyte imbalance	2.11	1.94, 2.30	<0.001	2.02	1.85, 2.20	<0.001
Cumulative number of electrolyte imbalances						
0	Ref	—		Ref	—	
1	1.38	1.27, 1.51	<0.001	1.39	1.27, 1.52	<0.001
2	2.60	2.37, 2.85	<0.001	2.54	2.31, 2.79	<0.001
3	2.69	2.47, 2.93	<0.001	2.55	2.32, 2.81	<0.001

## Discussion

Due to the lack of a precise definition, the true prevalence of RFS is not described in most published studies, and only focuses on hypophosphatemia [19]. Reported prevalence varies from 0-2% to 50-80% in systematic reviews and other case series [20]. Malnourished patients have a higher risk of hypophosphatemia, with mortality rates as high as 18.2% in severe cases, compared to 4.6% in those without malnutrition. Critically ill patients receiving TPN for ≥48 hours have a 34% probability of developing hypophosphatemia [21].

Patients with prolonged periods of minimal or no nutritional intake related to critical illness or major surgery are at high risk of developing RFS. The NICE criteria provide guidance on clinical nutritional status for critically ill and chronically ill populations at high risk for RFS [18,22]. Continuous monitoring is essential to identify early signs and symptoms of this condition, such as hypophosphatemia, hypokalemia, hypomagnesemia, selenium deficiency, glucose abnormalities, neurological changes such as delirium or seizures, arrhythmias, heart failure, and respiratory failure [23-25].

In this study, a total of 195 patients were included and analyzed during their stay in the ICU with TPN. The results obtained show a high prevalence of electrolyte imbalances, particularly hypokalemia (37%), hypophosphatemia (36%), and hypomagnesemia (24%). It is noteworthy that 37% of the patients developed RFS, a figure considerably higher than that reported in the pre-existing literature. This could be attributed to the specific clinical profile of the studied population, as well as to comorbidities and metabolic conditions prior to the initiation of TPN. Furthermore, it is important to mention that the severity of RFS in most patients did not have a significant clinical impact or produce serious complications and did not have an associated fatal outcome; in fact, the cause of death was due to other factors.

The most significant finding was a clear dose-response relationship between the cumulative number of electrolyte imbalances and the risk of developing RFS, with a progressive, statistically significant increase in risk. This finding suggests that not only does the presence of hypophosphatemia, hypokalemia, or hypomagnesemia alone represent a considerable risk, but their combination increases the likelihood of triggering RFS. This shows that having pot-TPN administration three or more associated imbalances doubles the risk, even after performing the corresponding multivariable adjustment. This reinforces the importance of close monitoring of electrolyte changes as a prevention strategy. In addition, the use of personalized supplemental nutrition can modify the development of RFS, as it is adapted to the needs of each patient and the underlying condition that led to their hospitalization, along with close monitoring in the ICU [15].

The study results are consistent with those reported in the literature by Friedli et al., which highlight hypophosphatemia as the sentinel marker of RFS [17]. However, the cumulative effect of multiple alterations on the development of RFS has not been specifically reported, which can be considered a highly relevant contribution of the present study. On the other hand, the high prevalence of nutritional risk (72% with NRS ≥5) coincides with previous findings in hospital populations, highlighting the need to strengthen and standardize systematic nutritional screening upon hospital admission, as well as continuous reassessment during their stay [1,2].

Early identification of electrolyte imbalances as a manifestation of RFS should be a priority in the ICU, since early diagnosis and preventive measures can prevent fatal complications such as arrhythmias or death. Furthermore, medical staff should be trained on this topic to raise awareness and incorporate an active monitoring plan to asses metabolic and biochemical safety of nutritional support. Likewise, reinforcing the routine use of supplemental regimens should be considered part of the primary strategies in these patients [15,18].

Regarding the study's limitations, it is important to note that it is a single-center study, which may limit the generalizability of the results. Furthermore, although multivariate adjustment was performed during the statistical analysis, variables related to liver function or nitrogen balance that could influence the occurrence of liver-related syndrome (LRS) were not included; also, by identifying frequent use of preformed TPN presentations, it provides variability due to the multiple presentations available and used in the hospital and there was also no objective and measurable assessment of sarcopenia, even though it is a very common nutritional condition in these patients. However, the inclusion of all available cases with well-defined inclusion criteria, as well as the analysis performed, strengthens the study's internal validity.

Finally, the study not only reinforces what has been described in the literature, but also proposes and expands clinical knowledge by demonstrating the cumulative effect of electrolyte imbalances in this condition. This highlights the urgent need to incorporate clinical protocols for screening and proactive prevention from the first 24-28 hours of nutritional support, as suggested by the guidelines of the American Society for Parenteral and Enteral Nutrition (ASPEN) [3] and the European Society for Clinical Nutrition and Metabolism (ESPEN) [7].

This original research provides concrete and scientifically rigorous evidence that supports institutional changes in nutritional support practices in other ICUs. Currently, there is no research in Mexico on this condition, and this study also raises new questions for future research, such as the role of other biomarkers such as albumin and pre-albumin, transferrin, C-reactive protein, and interleukins in the diagnosis and management of RFS.

## Conclusions

RFS is a common complication in critically ill patients receiving TPN. The presence of electrolyte imbalances is directly and significantly associated with the development of this syndrome, so identifying them early allows the establishment of preventive interventions to reduce morbidity and mortality associated with this nutritional support. 

The findings of this study reinforce the need to implement a systematic, standardized nutritional assessment for all hospitalized patients and to monitor TPN administration closely to reduce associated morbidity and mortality. The identification of the RFS should be mandatory and is a standard of quality of care in the ICU, as well as an indicator of safety and clinical excellence.
